# Vessel wall characterization using quantitative MRI: what’s in a number?

**DOI:** 10.1007/s10334-017-0644-x

**Published:** 2017-08-14

**Authors:** Bram F. Coolen, Claudia Calcagno, Pim van Ooij, Zahi A. Fayad, Gustav J. Strijkers, Aart J. Nederveen

**Affiliations:** 1 Department of Biomedical Engineering and Physics, Academic Medical Center, PO BOX 22660, 1100 DD Amsterdam, The Netherlands; 2 Department of Radiology, Academic Medical Center, Amsterdam, The Netherlands; 3 Translational and Molecular Imaging Institute, Icahn School of Medicine at Mount Sinai, New York, NY USA; 4 Department of Radiology, Icahn School of Medicine at Mount Sinai, New York, NY USA

**Keywords:** MRI, Atherosclerosis, Quantitative imaging, Vessel wall imaging, Plaque imaging

## Abstract

The past decade has witnessed the rapid development of new MRI technology for vessel wall imaging. Today, with advances in MRI hardware and pulse sequences, quantitative MRI of the vessel wall represents a real alternative to conventional qualitative imaging, which is hindered by significant intra- and inter-observer variability. Quantitative MRI can measure several important morphological and functional characteristics of the vessel wall. This review provides a detailed introduction to novel quantitative MRI methods for measuring vessel wall dimensions, plaque composition and permeability, endothelial shear stress and wall stiffness. Together, these methods show the versatility of non-invasive quantitative MRI for probing vascular disease at several stages. These quantitative MRI biomarkers can play an important role in the context of both treatment response monitoring and risk prediction. Given the rapid developments in scan acceleration techniques and novel image reconstruction, we foresee the possibility of integrating the acquisition of multiple quantitative vessel wall parameters within a single scan session.

## Introduction

Atherosclerosis is the leading cause of death in the western world, and is responsible for the majority of cerebrovascular and cardiovascular events such as ischemic stroke and myocardial infarction [1]. Atherosclerosis consists in the formation of “plaques” in the arterial vessel wall. Endothelial dysfunction, mainly related to local reduction of wall shear stress in the presence of non-laminar flow profiles, plays a pivotal role in the initiation and progression of atherogenesis. Disruption of the endothelial barrier facilitates the subendothelial accumulation of lipids, which triggers the initial inflammatory response that leads to plaque formation. Subsequent progression of atherosclerotic disease involves numerous processes that continuously alter vessel wall composition, including smooth muscle proliferation and angiogenesis, as well as the formation of intraplaque hemorrhage (IPH), lipid necrotic core and calcifications [2, 3].

Non-invasive imaging techniques have played an important role in the assessment of different plaque phenotypes, as well as in measuring changes in biological processes that occur during the different stages of atherosclerosis development [4]. Vessel wall magnetic resonance imaging (MRI) has proven to be a powerful technique for characterizing atherosclerosis in various regions of the vascular system, including the carotid and coronary arteries, aorta, and peripheral and intracranial arteries [5–10]. By enabling the evaluation of plaque composition and physiology, in vivo vessel wall MRI has helped refine the assessment of plaque risk profiles for rupture and subsequent cardiovascular events beyond simple lesion size [11, 12]. Initial studies used qualitative imaging primarily for identifying distinct patterns of high/low signal intensity associated with different phenotypes of atherosclerotic plaque. In the past few years, however, there has been increasing interest in developing imaging methods that provide quantitative data related to vessel wall structure and function. Not only does this improve longitudinal monitoring of the progression of atherosclerosis; it also provides sensitive disease markers that may serve as surrogate endpoints for evaluating the effect of novel treatment strategies.

Contrast between different plaque components may originate from differences in their relaxation time constants T1 and T2. These values can be quantified in each voxel by parametric fitting of several images with different contrast weighting. The resulting T1 and T2 maps enable automated plaque segmentation in its various constituents. Additionally, the MR signal can be made sensitive to water diffusion, which can be used to map the spatial variation in the apparent diffusion coefficient (ADC) within the plaque. This is of particular interest for detecting lipid accumulation, one of the major risk factors associated with plaque rupture. Contrast-enhanced MRI has been used to assess changes in vessel wall permeability, which can be increased both through disruption of the luminal endothelial layer and by the formation of leaky angiogenic vessels within the plaque. More specifically, imaging with dynamic contrast-enhanced (DCE) MRI, a technique that samples the influx of contrast agent in the plaque over time using fast T1-weighted (T1w) imaging sequences, has enabled the quantification of several pharmacokinetic parameters, including endothelial permeability and microvascular volume. Finally, blood flow measurements with phase-contrast MRI can be used to quantify pulse wave velocity (PWV), which is a common evaluation of vessel wall stiffness. More recently, 4D flow MRI has enabled local measurement of wall shear stress (WSS), which plays a crucial role in vascular endothelial function.

In this review, we present emerging techniques for quantitative MR imaging of the vessel wall. While the main focus will be on the carotid arteries, the most extensively studied vascular bed, examples in other vascular regions (aorta, intracranial vessels) will also be touched upon. We will present the latest developments in MR sequence and protocol design, and discuss their advantages and pitfalls in the quantification of vessel wall composition and function. While vessel wall thickness reflects anatomical rather than structural/functional information, it is still considered an important quantitative parameter in characterizing atherosclerotic burden. We therefore start with a short overview of different two- and three-dimensional sequences that are used for this purpose, which are often the basis for other quantitative methods as well. Finally, we will present current promising developments in MRI that will allow further improvement in the techniques presented here.

## Plaque burden

Vessel wall thickening is one of the early visible manifestations of atherosclerosis, and therefore remains one of the most important diagnostic readouts of atherosclerotic burden. Large clinical studies have demonstrated an association between carotid intima-media thickness as measured with ultrasound, and overall risk for cardiovascular events such as stroke or myocardial infarction [13, 14]. Ultrasound still remains the first choice in clinical practice for assessing carotid stenosis after ischemic events, not least for its cost effectiveness. However, black-blood MRI techniques for measuring plaque burden have improved tremendously in recent years, and are increasingly used in studies on atherosclerosis progression or treatment effect [15–17]. Moreover, MRI has no limitations in depth penetration and is thus a powerful tool for investigating not only superficial arteries such as the carotids, but also those such as the intracranial [18] and coronary arteries [7].

### 2D black-blood MRI

Blood suppression is essential for achieving accurate delineation of the vessel wall, which would otherwise be compromised by smearing of the bright-blood lumen signal. Two-dimensional (2D) T2-weighted (T2w) spin-echo sequences have inherent blood suppression due to outflow effects at long echo times; however, this mechanism is not compatible with short echo times needed for T1w imaging.

**Fig. 1:**
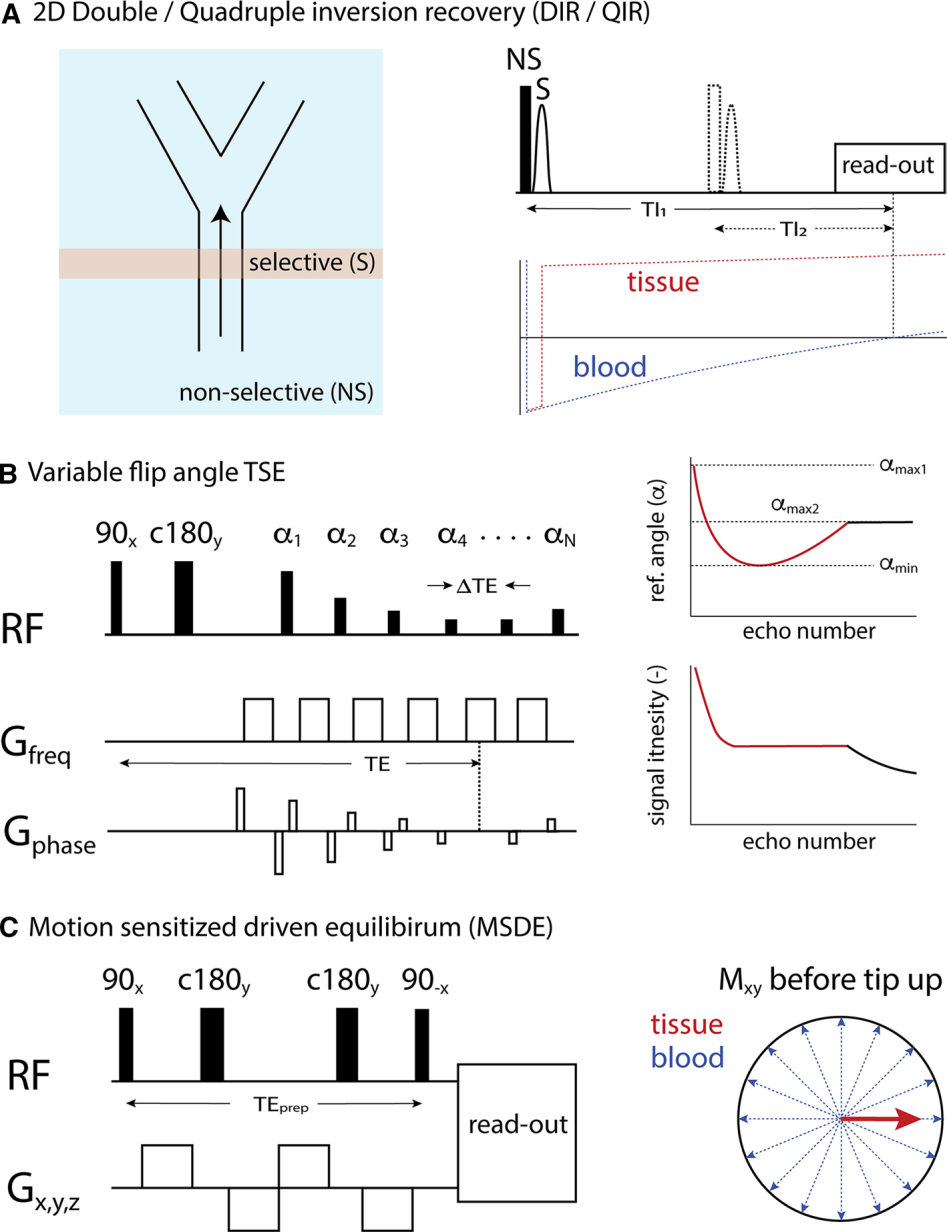
2D/3D blood suppression techniques. **a** 2D double/quadruple inversion recovery. Non-selective inversion of inflowing blood generates black-blood at a correctly timed inversion time. **b** 3D variable flip angle TSE. Frequency encoding gradients within the TSE readout have flow suppression properties in that direction. The point-spread function of the acquisition can be improved by variable flip angle schemes. **c** 3D motion-sensitized driven equilibrium (MSDE). Within a T2 preparation module, strong gradients dephase moving spins within a voxel, effectively crushing the blood signal. Magnetization of static tissue, although slightly T2-weighted, is restored by a final tip-up pulse. Subsequently, a 3D TFE/TSE readout can be applied

The first robust technique allowing for 2D T1w black-blood imaging of the arterial vessel wall was proposed by Edelman et al. [19]. This spin-echo-based method achieves blood suppression by using a pair of non-selective and slice-selective inversion pulses (double-inversion recovery axial image recovery, or DIR), thereby effectively inverting only the tissue and blood outside the imaging slice (Fig. 1a). The inversion time (TI) between the inversion pulses and imaging readout is chosen such that blood longitudinal magnetization is nulled (as a result of T1 relaxation), while at the same time non-inverted blood flows out of the imaging slice. The slightly higher signal-to-noise ratio (SNR) and robust blood suppression make T1w imaging the preferred choice for 2D vessel wall thickness measurements.

Unfortunately, the dependency on blood outflow from the imaging slice renders a multi-slice or three-dimensional (3D) implementation ineffective [20]. Another problem obviously arises when post-contrast measurements are performed, where the T1 of blood decreases significantly, and the optimal TI is difficult to determine beforehand. Yarnykh, however, has proposed an elegant solution by introducing a second inversion pair (quadruple inversion recovery, or QIR), making blood suppression effective over a much larger range of T1 values [21].

### 3D black-blood MRI

Increasing interest in isotropic 3D imaging protocols has led to the development of 3D black-blood imaging sequences that do not rely on outflow effects and are therefore more robust against slow flow artifacts. 3D turbo spin-echo (TSE) sequences actually have inherent black-blood properties themselves, caused by the buildup of intravoxel dephasing as a result of the positive gradient moment of the readout gradient during the echo train. While this was presented in the early literature as an alternative to bright-blood brain angiography [22], successful implementation for vessel wall imaging required novel variable-flip-angle (VFA) refocusing schemes [23–25] that also allowed a stable signal response for longer echo trains (Fig. 1b). This ensures a favorable point-spread function to prevent blurring of the thin vessel wall. Naturally, this can only be achieved for specific values of T1 and T2, and the exact choice of flip angle scheme will always be a trade-off between tissue contrast and effectiveness of flow suppression.

Another class of black-blood methods achieves blood suppression independent of the acquisition scheme through the use of black-blood preparation modules. One method, motion-sensitized driven equilibrium (MSDE, Fig. 1c), combines T2 preparation with flow-sensitizing gradients [26, 27]. If the first moment of the preparation module is sufficiently high, intravoxel dephasing for flowing blood occurs, while the static tissue effectively only undergoes T2 relaxation during the time interval TE_prep_ .

The final tip-up pulse restores the static tissue longitudinal magnetization for subsequent readout using turbo field-echo (TFE) or TSE acquisition schemes. As shown by Fan et al. [23], the latter seems the more effective strategy, as it adds the black-blood properties of the TSE readout.

An alternative preparation method uses so called-DANTE (delay alternating with nutation for tailored excitation), consisting of a non-selective train of small-flip-angle RF pulses [28, 29]. While this preparation is much longer than MSDE (~100–150 ms), static tissue signal is better preserved and suppression occurs even at low velocities. DANTE is particularly promising for intracranial imaging, where it also enhances suppression of cerebrospinal fluid closely surrounding the vessels [30–33].

### 2D versus 3D imaging

**Fig. 2:**
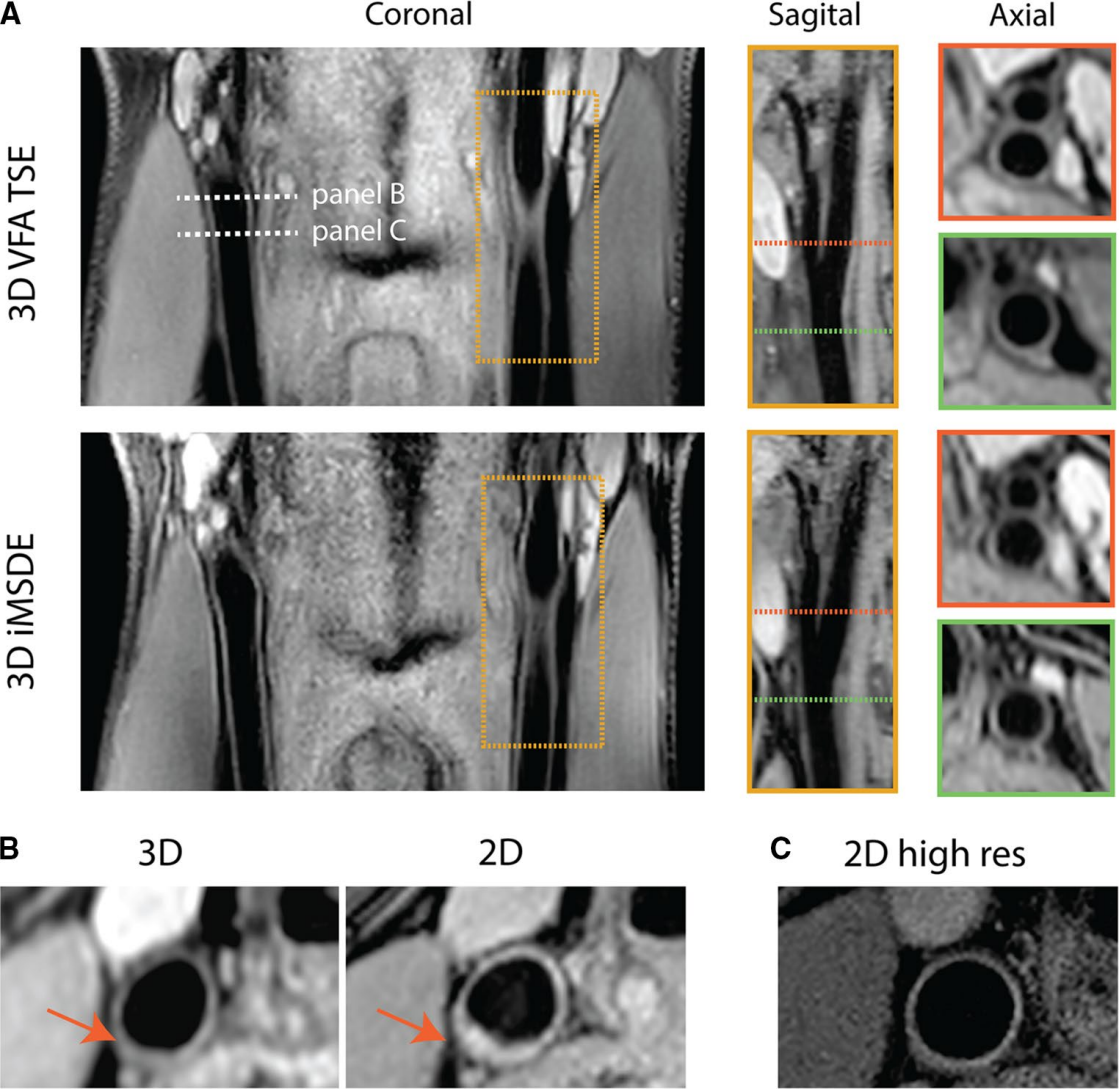
Vessel wall imaging using 2D and 3D imaging sequences. Sequence parameters: **a** FOV = 144 × 144 × 35 mm, resolution = 0.7 × 0.7 × 0.7 mm. *3D MSDE*, TR/TE = 10.0/3.5 ms, TE_prep_ = 11.5 ms, TFE factor = 60, Acq time = 3 min. *3D VFA TSE*, TR/TE = 1000/28 ms, ∆TE = 4.3 ms, TSE factor = 40, start-up echoes = 4, Acq time = 6 min. **b** *3D TSE*, same as (a), *2D DIR*, ST = 2 mm, resolution = 0.5 × 0.5 mm, TR/TE = 1000/3.5 ms, TI = 400 ms **c** *2D DIR*, same as (b), but with resolution = 0.25 × 0.25 mm

Figure 2a shows examples of 3D MSDE TFE and VFA TSE carotid scans in the same volunteer, illustrating the ability to acquire black-blood coronal images covering the common and internal carotid arteries. Because of the isotropic voxels, these data can easily be reformatted into sagittal and axial views. Figure 2b clearly illustrates that 2D compared to 3D imaging may result in overestimation of wall thickness in regions near or within the bifurcation, where the vessel diameter changes quickly. On the other hand, we also know that in locations with less curvature, ECG-gated 2D DIR scans with in-plane resolution of 0.25 mm have optimal vessel wall delineation, as shown in Fig. 2c. These measurements were validated against histology and ultrasound in a pig model [34], and resulted in a mean carotid vessel wall thickness of 0.49 mm [35].

Given the current resolution in 3D MRI protocols, overestimation of vessel wall thickness is likely to occur [36]—especially considering smaller vessels such as the intracranial or coronary arteries [37, 38]—although this issue may be less problematic in later stages of atherosclerosis, where plaque formation has caused significant wall thickening [39].

While excellent reproducibility values for 2D thickness measurements have been reported for carotid and aortic vessel walls [40, 41], a great advantage of 3D imaging is that it requires no tedious, accurate planning of the imaging slices, while still allowing reconstructions in arbitrary planes. This may partly explain the good reproducibility of 3D black-blood methods reported for various applications, such as the carotid arteries [42, 43], thoracic aortic wall [8, 24, 44], abdominal aorta [44] and even the coronary arteries [38]. While generally not applied, ECG and/or respiratory triggering can further minimize blurring due to vessel wall pulsation or breathing motion [8].

Although different black-blood mechanisms can be easily described, their exact performance in terms of SNR, tissue/lumen contrast-to-noise ratio (CNR), and effective resolution are highly dependent on the exact sequence parameters, including both the preparation module and the specific acquisition scheme (e.g. TFE vs. TSE).

While comparisons between different methods have been reported [29], the large number of sequence parameters makes a fair comparison very challenging. Consequently, no consensus on optimal vessel wall imaging protocols has been reported thus far.

## Plaque composition

The excellent intrinsic soft tissue contrast of MRI has enabled visualization of different structural components within the plaque, such as lipid-rich necrotic core (LRNC), calcification (CA), fibrous tissue (FIB) and intraplaque hemorrhage (IPH). To this end, many studies have used multi-contrast-weighted imaging, in which each component can be identified by the specific combination of hypo- and/or hyperintense signal intensities on T1-, T2^(*)^ - and protein density-weighted (PDw) images [6, 45, 46]. Although accumulating evidence from imaging studies shows that the presence or absence of these components can be related to subsequent cardiovascular events [11, 47], qualitative interpretation of these images or the need to calculate relative signal intensities (e.g. compared to sternocleidomastoid muscle) gives rise to high intra/inter-observer variability [48]. This is mainly due to the strong influence of spatial variations in coil sensitivity on relative signal intensity, as well as the specific choice of MR sequence parameters. Therefore, the quantification of T1 and T2^(*)^ relaxation time constants of tissue on a voxel-wise basis—as quantitative measures of the underlying tissue composition—is of great interest. While quantitative T1 and T2^(*)^ values have been reported in histological studies of carotid endarterectomy specimens [49, 50], translation of existing techniques to in vivo vessel wall imaging has long been complicated by the additional need for high-resolution imaging, blood suppression and cardiac gating. The following sections will present the newest techniques that have overcome most of these challenges.

### T1 and T2^(*)^ relaxometry

**Fig. 3:**
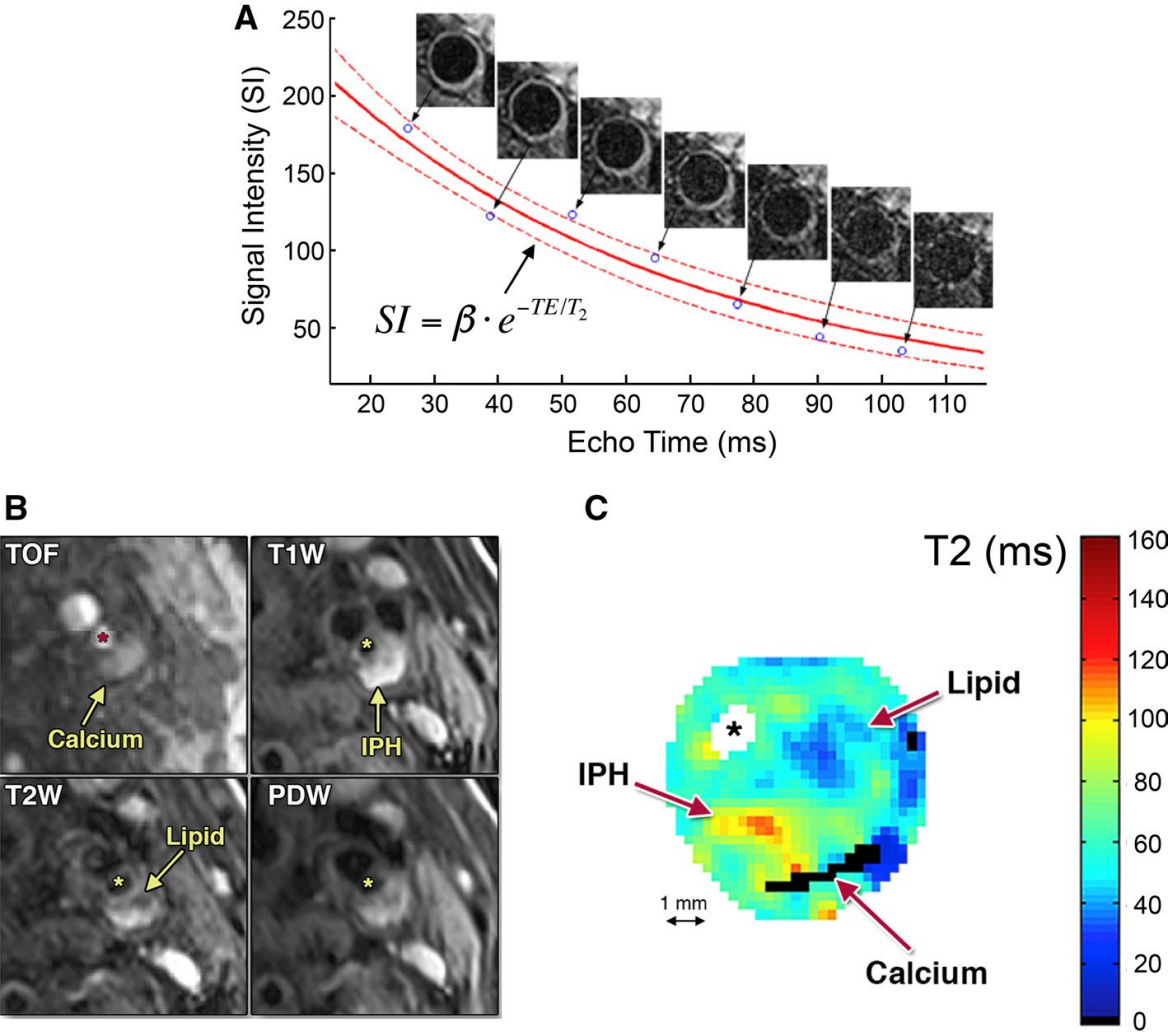
**a** Quantitative vessel wall T2 mapping. ECG-gated multi-echo spin-echo data are acquired with echo times between 25–100 ms. Blood suppression is obtained through double-inversion recovery preparation. **b** T2w image along with quantitative T2 map of an advanced plaque (*SM* smooth muscle, ^*^ lumen). Semi-automated segmentation of plaque components from the T2 map is shown on the right. Adapted from Biasiolli et al. (19) and Chai et al. [66]

Results from multi-contrast imaging studies show that LRNC has lower T2 than healthy intima/media. Although IPH may show large changes in T2 over time [51], the most commonly found “recent IPH” is associated with elevated T2 values. A traditional way of measuring T2 is by exponential fitting of signal intensities acquired with multi-echo spin-echo sequences (Fig. 3a). While the early literature had already reported in vivo vessel wall T2 quantification from dual spin-echo sequences [52], Biasiolli et al. have only recently improved this approach, allowing for acquisition of multiple echoes between 25 and 100 ms [53]. Since the need for relatively short echo times reduces the outflow effect compared to regular T2w spin-echo sequences, blood suppression was enhanced using DIR preparation (Fig. 1a). Figure 3b nicely illustrates a T2w image of an advanced plaque, along with corresponding quantitative T2 maps. In this study, the mean values for FIB and LRNC were 56 ± 9 and 37 ± 5 ms, while various patches of IPH had T2 values >90 ms. By training a Bayes classifier using expert readings of co-registered multi-contrast data, the authors developed an automated segmentation algorithm that discriminates the various plaque components based on their absolute T2 values. A significant drawback of the method is that it is limited to 2D imaging, with a relatively long acquisition time of 320 R–R intervals despite the use of 5/8 partial Fourier acceleration. Nonetheless, these results show that all relevant plaque components may be discriminated on the basis of T2 alone.

Instead of using multi-echo spin-echo-based sequences, T2* can be determined using a blood-suppressed multi-echo gradient echo approach, with typical echo times of 3–40 ms. Unlike T2, T2* is strongly affected by the presence of magnetic field inhomogeneities and thus might not solely reflect tissue structure. At the same time, this makes T2* imaging very sensitive in detecting protein-bound iron. Raman et al. [54] were the first to conduct an extensive study of the role of iron in atherosclerosis using quantitative T2* measurements. They found a significant decrease in T2* between asymptomatic (34.4 ± 2.7 ms) and symptomatic patients (20.0 ± 1.8 ms). Furthermore, ex vivo iron quantification in endarterectomy specimens showed equal total iron content in both groups, but greatly reduced levels of paramagnetic Fe(III) complexes. Overall, these results strongly suggest that symptomatic plaques are associated with higher amounts of ferritin-bound iron, which was sensitively assessed using quantitative T2* MRI. In addition to endogenous iron, T2*-weighted imaging has also been frequently used to detect macrophage-mediated uptake of intravenously injected superparamagnetic iron oxide (SPIO) particles as a surrogate marker of plaque inflammation [55, 56]. However, such studies still suffered from the use qualitative MRI methods [57]. In more recent studies, quantitative T2* mapping has been applied in combination with QIR blood suppression in order to increase the accuracy in assessing SPIO accumulation by calculating ΔT2* or ΔR2* between pre- and post-contrast scans [58, 59]. While T2* values can be prone to magnetic field inhomogeneities, both studies did report similar baseline T2* values of approximately 25 ms, indicating good reproducibility with this approach. Unfortunately, 3D implementation of these multi-echo techniques has not yet proven feasible, most likely due to the inevitable increase in repetition time, leading to clinically unacceptable acquisition times.

**Fig. 4:**
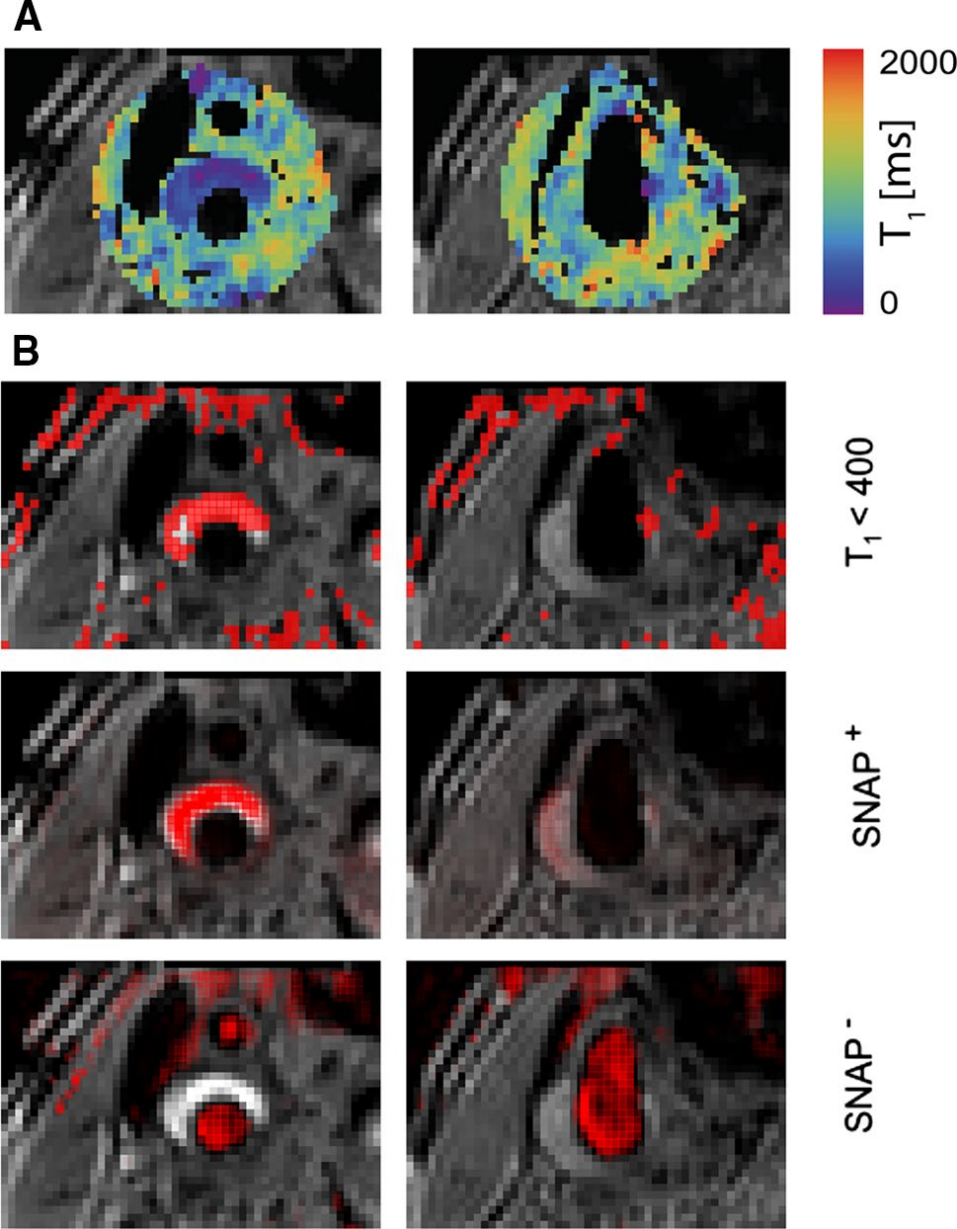
**a** Quantitative 3D vessel wall T1 mapping. The slice on the *left* shows regions of greatly reduced T1, indicating IPH. Adapted from Coolen et al. [31] **b** Masked images corresponding to *panel* **a** showing regions with T1 <400 ms, alongside registered SNAP images showing regions of IPH (SNAP^+^) or arterial blood (SNAP^−^). Regions with low quantitative T1 values match IPH-positive regions from SNAP (unpublished data)

T1 mapping methods are well validated in areas such as brain or cardiac imaging, and in these areas they are generally based on steady-state imaging at multiple flip angles or sampling of the inversion recovery longitudinal relaxation curve [60, 61]. Unfortunately, these are all bright-blood techniques that compromise vessel wall delineation and are susceptible to flow artifacts. We recently developed a black-blood version of the DESPOT1 approach [60] by using MSDE blood suppression with short pre-pulses of 11.5 ms and maintaining steady-state conditions of the 3D RF spoiled gradient echo train by adding dummy pulses after signal acquisition [62]. This enabled 3D vessel wall T1 mapping with isotropic resolution of 0.7 mm. Moreover, T2 mapping was possible using the same sequence by varying the TE_prep_ time of the MSDE pre-pulse. While good reproducibility of this 3D approach was shown, both carotid T1 and T2 values (844 ± 96 ms/39 ± 5) were lower than with single-slice TSE-based measurements (1227 ± 47/55 ± 11 ms) [63]. Furthermore, the use of MSDE pre-pulses causes T1 modulation in k-space, which can lead to some blurring of the resulting T1 maps. Figure 4a shows quantitative T1 mapping results from a carotid plaque where significantly reduced T1 is indicative of IPH. Simultaneous non-contrast angiography and intraplaque hemorrhage (SNAP) is a recently published method for detecting IPH, which can identify regions of IPH by phase-sensitive reconstruction of inversion recovery data [64]. Figure 4b shows SNAP^+^ and SNAP^−^ images corresponding to the region shown in Fig. 4a, representing IPH and arterial blood, respectively. Since the sign of the SNAP signal changes below a specific T1 value, we can show that the IPH region visible in the SNAP^+^ image can be recreated from the quantitative T1 map by simple thresholding.

While the main benefit in using quantitative T1 and T2^(*)^ mapping—compared to multi-contrast T1w and T2^(*)^w imaging—is to improve reproducibility and facilitate longitudinal monitoring of changes in plaque composition, it still does not allow for direct assessment of the relative contribution of different tissues in each voxel. In contrast, for instance, Koppal et al. were able to assess quantitative maps of fat content based on Dixon imaging, showing significant differences between the lipid core (12.6%) and surrounding tissue (9.2%) [65]. On the other hand, the use of multi-contrast imaging to detect different plaque components (i.e. LRNC, IPH) has been well validated against histology [45, 51]. Quantitative relaxation parameter mapping extends this concept and might enable better definition of thresholds for discriminating between these different tissue types. Indeed, a recent study reported that LRNC detection based on T2 mapping—pixels with T2 <42 or T2 >90 ms when IPH was included—showed very good correlation with histology (*R* = 0.85) and had good sensitivity (AUC = 0.79) for detecting recently symptomatic plaques [66].

### Diffusion

MRI is also able to quantify water diffusion within tissues. Strong field gradients applied on each side of a 180° refocusing pulse cause diffusion-mediated signal attenuation due to phase dispersion of spins. Conversely, in static tissue, the effect of both gradients cancels out and the signal is maintained. The degree of diffusion weighting is given by the b-value, which depends on the gradient strength, duration and spacing [67]. Similar to varying TE to quantify T2^(*)^, the apparent diffusion coefficient (ADC) can be estimated by an exponential fit of signals acquired at different b-values, which determines the amount of diffusion weighting. In vessel wall imaging, diffusion weighting is of particular interest for detecting the presence of an LRNC, which from ex vivo studies has been known to have a strongly decreased ADC [68, 69]. In fact, Clarke et al. showed that, compared to T1 and T2 quantification, ADC was the parameter that could best distinguish LRNC from FIB [70].

Diffusion-weighted imaging (DWI) is typically performed using 2D single-shot echo-planar imaging (EPI) sequences, which provides time efficiency for sufficient averaging of the low DW signal (because of the additional need for long TEs). However, EPI is very susceptible to B_0_ inhomogeneities, because phase errors accumulate for each additional phase encoding step. Kim et al. [71] were the first to apply inner-volume imaging for vessel wall applications, reducing the effective field of view (FOV) and thereby the echo train length by a factor of 4. In this way, good-quality ADC maps of 2-mm slices were obtained with a resolution of 1 × 1 mm^2^. This spatial resolution, however, is still inferior to the resolution used for vessel wall thickness measurements.

**Fig. 5:**
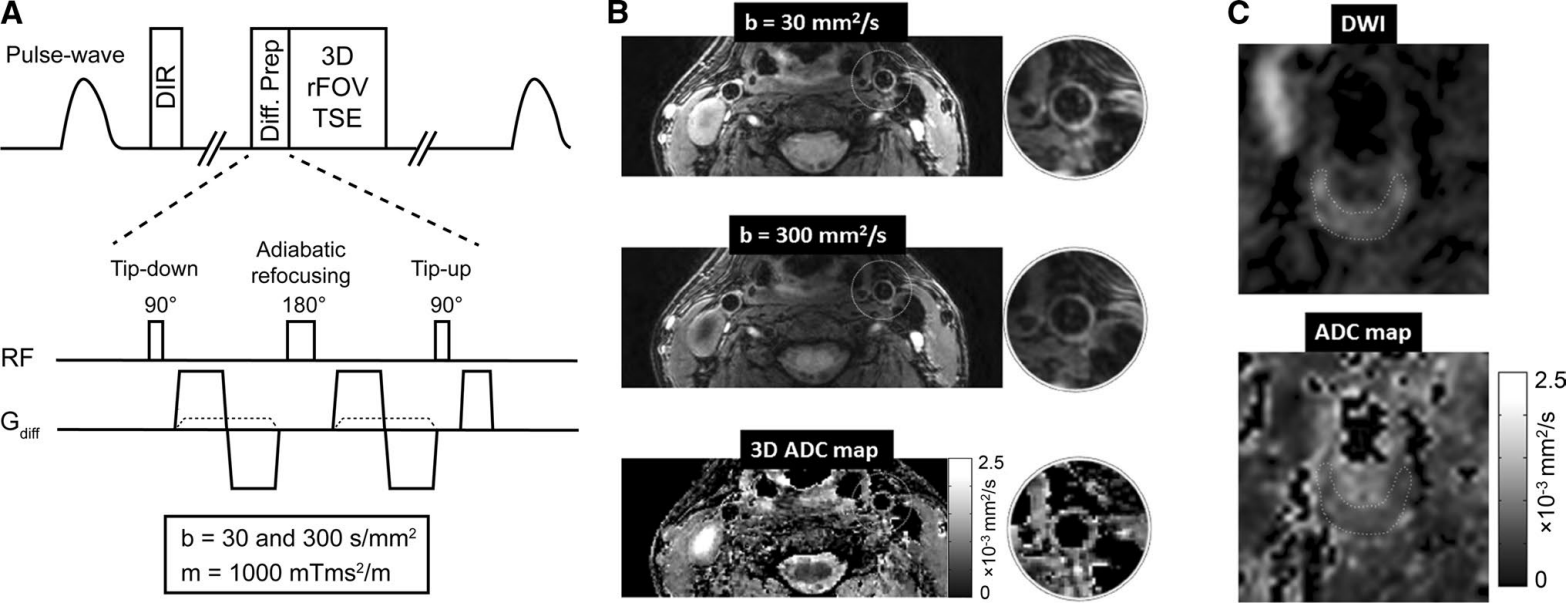
3D quantitative diffusion vessel wall imaging **a** ECG-gated 3D diffusion-prepared variable-flip-angle TSE sequence. Motion-compensated diffusion gradients are combined with low b-value flow-sensitizing gradients for additional blood suppression. **b** Diffusion-weighted vessel wall images of a healthy volunteer at *b* values of 30 and 300 mm^2^/s, along with corresponding quantitative ADC map. **c** Diffusion-weighted imaging of a lipid-rich atherosclerotic plaque with high signal on the *b* = 300 mm^2^/s image and corresponding low ADC values. Adapted from Xie et al. [72]

A novel approach enabling 3D vessel wall DWI was recently presented by Xie et al. [72]. They decoupled diffusion weighting from the imaging readout through the use of a motion-compensated diffusion-weighted pre-pulse (Fig. 5a). Combined with 3D TSE acquisitions, this resulted in high-resolution imaging of 0.6 × 0.6 × 2 mm^3^ (Fig. 5b). Additional double-inversion recovery preparation and low-b-value motion-sensitized gradient on top of diffusion weighting further guaranteed efficient blood suppression. A large decrease in LRNC ADC values compared to FIB (0.6 × 10^−3^ vs. 1.27 × 10^−3^ mm^2^/s) was shown, indicating that ADC mapping may serve as a sensitive and quantitative non-contrast technique for plaque characterization (Fig. 5c). Using a similar approach but with a stimulated echo pathway, Zhang et al. [73] recently showed that phase errors arising from eddy currents could be prevented. This resulted in stable carotid artery ADC values across subjects of 1.4 ± 0.23 × 10^−3^, which is comparable to results of earlier studies.

While challenging, extension of DWI to a diffusion tensor imaging (DTI) protocol using multiple gradient directions would allow calculation of the fractional anisotropy (FA) of the vessel wall microscopic fiber structure. Opriessnig et al. [74] were recently the first to apply a 2D DTI sequence using four b-values and 18 diffusion directions on a 10-mm carotid artery segment. In 12 healthy volunteers, the authors found a significant correlation between FA and age, indicating possible alterations of the vessel wall microstructural integrity. Moreover, the reproducibility of FA measurements appeared very high, with CV values no higher than approximately 5%.

## Quantification of permeability with dynamic contrast-enhanced MRI

**Fig. 6:**
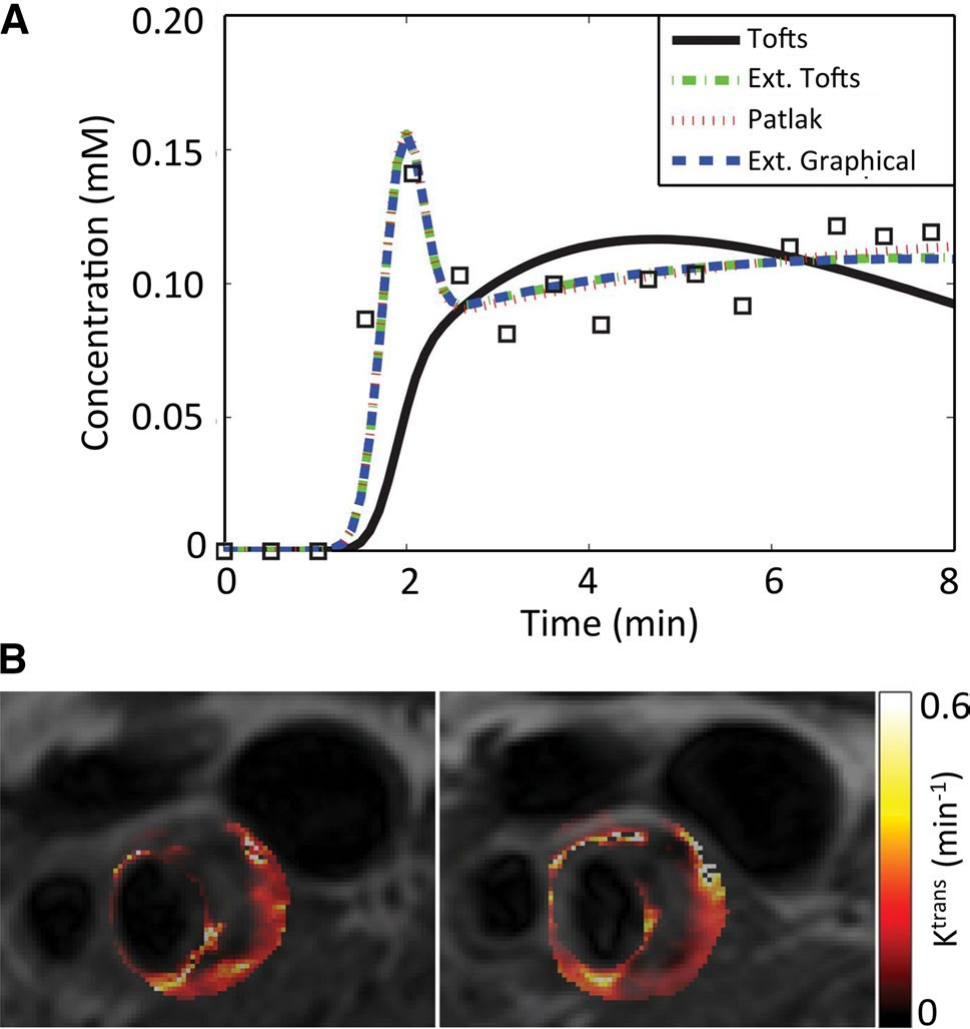
**a** Representative concentration versus time curve kinetics in atherosclerotic plaques. Data fitting is shown with 4 different models. *Ext.* extended. **b** Example of pixel-by-pixel parametric *K* ^trans^ maps of the carotid arteries, calculated using a Patlak model. Both maps were generated from the same individual and from images acquired 1 week apart, in order to evaluate inter-scan reproducibility. Adapted from Gaens et al. [79]

Inflammation in vulnerable atherosclerotic plaques, at high risk for causing severe, acute cardiovascular events, is accompanied by the proliferation of existing and new microvessels with high endothelial permeability [75]. Dynamic contrast-enhanced (DCE) MRI, a technique widely used to quantify endothelial permeability and microvascular volume in tumors [76], has been widely adopted in the past 15 years for quantification of these parameters in atherosclerotic plaques as well. DCE-MRI consists in the rapid serial acquisition of T1-weighted MR images of a volume of interest while a T1-shortening, gadolinium (Gd)-based contrast agent is injected [76]. During imaging, the contrast agent extravasates from the plasma compartment, and causes MR signal enhancement in permeable tissues. Tissue microvascular volume and permeability can then be extracted from the kinetics of tissue signal enhancement over time. To calculate these quantities, MR signal enhancement curves are first converted to contrast agent concentration values, either by estimating the T1 relaxation time at each dynamic time frame, or by assuming a linear relationship between MR signal intensity and contrast agent concentration. After conversion to concentration, pixel-by-pixel or region of interest (ROI) concentration curves are typically analyzed using either model-based or non-model based approaches to calculate tissue properties (Fig. 6a), such as microvascular volume, permeability (Fig. 6b) and extravascular extra-cellular space volume. The most common kinetic models used for analysis of DCE-MRI are typically based on the modified Tofts model [77]:

$$C_{\text{tissue}} (t) = v_{\text{p}} \cdot C_{\text{p}} (t) + K^{\text{trans}} \int\limits_{0}^{t} {C_{\text{p}} (t') \cdot e^{{ - K^{\text{trans}} /v_{\text{e}}\cdot(t'-t)}} {\text{d}}t'},$$

where *C*_tissue_ (*t*) is the concentration of contrast agent in the tissue of interest, *C*_p_ is the contrast agent concentration in the plasma compartment, *v*_p_ (%) is the fractional microvascular volume, *K* ^trans^ indicates the permeability (min^−1^), *v*_e_ (%) is the extravascular extracellular space fraction, and *t* is time. In atherosclerosis, this model has often been used under the Patlak assumption [78, 79], which assumes no contrast agent “backflow” from the tissue to the plasma compartment, as shown below:

$$C_{\text{tissue}} (t) = v_{\text{p}} \cdot C_{\text{p}} (t) + K^{\text{trans}} \int\limits_{0}^{t} {C_{\text{p}} (t')\;{\text{d}}t'} .$$

In the literature, average plaque *K* ^trans^ values calculated using this model have ranged from 0.05 to 0.3 min^−1^ [80–88], while average *v*_p_ values were found to vary between 4 and 25% [78, 83, 85, 87, 89]. These broad ranges may reflect differences in patient populations and/or disease stages in animal models, or different acquisition or analysis methods. Using a 2D bright-blood (i.e. allowing sampling of the MR signal in the blood plasma during the dynamic acquisition) spoiled gradient recalled echo (SPGR) MR sequence and Patlak kinetic analysis, Kerwin et al. [78] were the first to demonstrate a significant, positive correlation between microvessel density in human carotid atherosclerotic plaques (CD31 immunostaining) and the parameter *v*_p_ (fractional microvascular volume) derived from DCE-MRI. Using this methodology, the same group also demonstrated a significant, positive relationship between *v*_p_, *K* ^trans^ (permeability) and plaque macrophages, neovasculature and loose matrix (LM) [83]. As for clinical parameters, *K* ^trans^ was found to correlate with lower levels of high-density lipoproteins (HDL) [83] and higher levels of C-reactive protein [80], and was higher in smokers than non-smokers [80, 83]. This analysis was extended to quantify the difference in DCE-MRI parameters between different plaque components including LRNC, IPH, LM, FIB and CA [89]. It was demonstrated that while LM and FIB showed relatively high values of *K* ^trans^ and *v*_p_, NC, IPH and CA exhibited significantly lower *K* ^trans^ and *v*_p_. O’Brien et al. [85, 90] recently demonstrated a relationship between the duration of statin therapy and *v*_p_ from DCE-MRI: the shorter the duration of statin therapy, the higher the *v*_p_ values. The presence of metabolic syndrome, higher body mass index and plasma lipoprotein(a) values were also associated with higher *v*_p_ values.

While the Patlak model has been widely used for quantifying endothelial permeability and microvascular volume in atherosclerosis, the best choice of model for analyzing DCE-MRI data of the vessel wall is still a topic of investigation [79, 91]. As an alternative to kinetic modeling, non-model-based approaches, such as area under the enhancement curve (AUC), uptake slope, time to peak or maximum concentration, can also be used to analyze DCE-MRI data. While the relationship between these non-model based parameters and microvascular volume and permeability is not straightforward, AUC has been particularly valuable as a surrogate measure of plaque neovascularization and permeability calculated from so-called black-blood DCE-MRI data, where the MR signal from the blood plasma is purposively suppressed to improve vessel wall delineation, and kinetic modeling cannot be easily performed. Using black-blood DCE-MRI, Calcagno et al. [92] demonstrated a positive, significant correlation between the parameter AUC and plaque microvessels count (CD31 immunostaining) in aortic plaques of atherosclerotic rabbits. The reproducibility of this technique was also evaluated and was found to be very good [93]. In addition, AUC has been used as a surrogate marker of drug efficacy to evaluate the impact on vascular permeability/inflammation of several approved (atorvastatin [94], pioglitazone [95]) and novel (liposomal corticosteroids [96], liver X receptor [LXR] agonist [94]) drugs. Chen et al. [97] showed an increase in both plaque *K* ^trans^ relative to skeletal muscle [98] and AUC in aortic plaques of atherosclerotic rabbits between 3 and 6 months of an atherosclerotic diet. AUC was also used by Calcagno et al. [99] to compare perfusion/permeability by DCE-MRI to vascular inflammation by ^18^F-fluorodeoxyglucose (FDG) uptake by positron emission tomography with computed tomography (PET/CT) in sub-clinical plaques of patients with risk factor for coronary artery disease (CAD). In this case the authors found a weak negative relationship between the two techniques in this patient population.

**Fig. 7:**
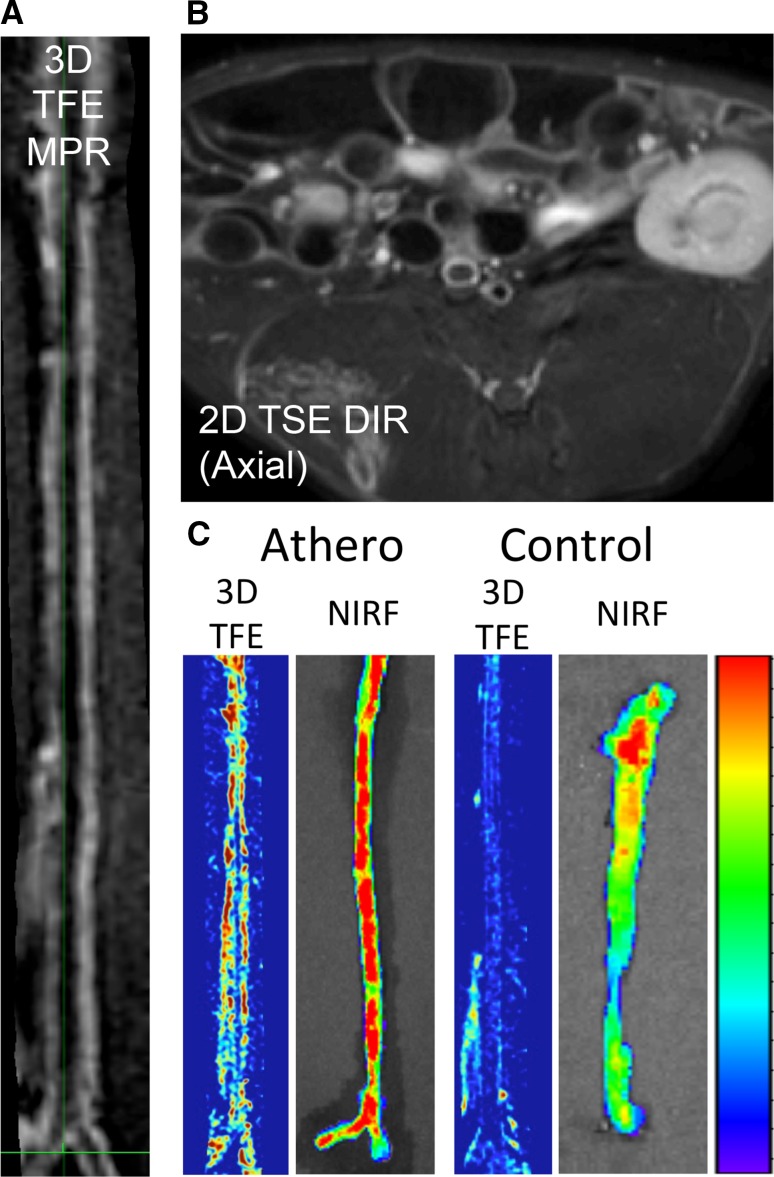
3D vessel wall imaging. **a** Multiplanar reconstruction (MPR) of 3D images acquired after the injection of Gd-DTPA into the aorta of an atherosclerotic rabbit. *TFE* turbo field echo. **b** Representative 2D turbo spin-echo (TSE) double-inversion recovery axial image acquired after injection of Gd-DTPA into the aorta of an atherosclerotic rabbit. **c** Corresponding AUC permeability maps (MPR) from 3D TFE DCE-MRI and near-infrared fluorescence (NIRF) after Evans blue (EB) injection in one representative atherosclerotic and control rabbit. Prominent uptake of MR contrast and EB is shown in the diseased animals compared to the control. Adapted from [86]

Since these initial applications, several significant developments have occurred in DCE-MRI of atherosclerosis. For example, both bright- and black-blood studies described above primarily employed 2D single- or multi-slice imaging with high spatial and temporal resolution, but offered limited coverage along the vascular bed examined. More recent studies have improved upon this aspect [86, 88, 100–102], and proposed the use of 3D isotropic high-resolution vessel wall imaging with extensive coverage for vascular DCE-MRI. Using a bright-blood 3D gradient recalled echo (GRE) approach, Taqueti et al. [100] demonstrated a positive, significant relationship between permeability by DCE-MRI and ^18^F-FDG PET/CT in patients with advanced carotid disease. The authors also confirmed a positive, significant relationship between *K* ^trans^ and microvessels (CD31 immunostaining) and inflammation (CD68 and major histocompatibility complex II [MHCII] immunostaining) in the same patient population. Similarly, van Hoof et al. [88] demonstrated a correlation between the vessel wall and adventitial *K* ^trans^ and plaque microvessels by histology in patients with carotid atherosclerosis. Using black-blood DCE-MRI instead, Kim et al. [103], Lobatto et al. [102] and Calcagno et al. [86] were able to quantify endothelial permeability in the whole abdominal aorta of atherosclerotic rabbits. These findings were confirmed by a positive, significant relationship between AUC by 3D DCE-MRI and endothelial permeability by ex vivo near-infrared fluorescence using fluorescent albumin or Evans blue dye (Fig. 7).

Despite these significant advances, vascular DCE-MRI is still significantly challenged in achieving accurate quantification of plaque microvascular burden and permeability. As mentioned above, it is difficult to extract fully quantitative information from black-blood vascular DCE-MRI (either 2D or 3D), due to the inability to sample the concentration of contrast agent in the blood plasma (the so-called arterial input function, AIF), which is a necessary input for kinetic models. On the other hand, even bright-blood vascular DCE-MRI approaches may carry some degree of error in the quantification of vascular permeability parameters, also stemming from potential inaccuracies when estimating the AIF from the MR signal itself. As with all other vessel wall imaging techniques, vascular DCE-MRI requires imaging with high spatial resolution, which may render the temporal resolution of the acquisition inadequate for sampling the fast contrast agent kinetics in the blood plasma. In addition, MR sequence parameters used for DCE-MRI are typically optimized to accurately capture the dynamic signal range of enhancement in atherosclerotic plaques, and may not be adequate to accurately capture signal enhancement in the vessel lumen, where contrast agent concentrations are much higher, and T1 much lower, during dynamic imaging. Recent studies have focused on overcoming these challenges by proposing either 2D [104] or 3D [105, 106] sequences that allow for accurate sampling of both blood and plaque kinetics, interleaving the acquisition of images with different spatial and temporal resolution and different imaging parameters. For example, AIF images can be acquired with lower spatial resolution, which allows for faster imaging (high temporal resolution) and imaging parameters optimized for the high signal enhancement (low T1 values) found in the vessel lumen. Conversely, plaque dynamic images can be acquired with high spatial resolution, lower temporal resolution, and imaging parameters optimized to capture the signal enhancement of the arterial vessel wall. Other authors have instead explored the use of phased-based rather than magnitude-based AIFs for kinetic modeling of vascular DCE-MRI data [107]. Using simulations and phantom experiments, the authors found that phase-based AIF offered a more accurate sampling of the true contrast agent kinetics in the blood plasma. While the absolute value of kinetic parameters derived from magnitude- and phase-based AIF were different, they were shown to be highly correlated.

### Alternative techniques for measuring permeability

In addition to DCE-MRI, various other techniques have been proposed for quantifying endothelial permeability in atherosclerotic plaques. Delayed-enhancement imaging with either low molecular weight gadolinium chelates or albumin-binding agents has been used as a semi-quantitative measure of plaque permeability in both animal models (mice [108], rabbits [109–111]) and humans [112, 113]. Phinikaridou et al. [114] and Bar et al. [115] quantified endothelial permeability in the brachiocephalic artery of atherosclerotic mice as a change in the vessel wall relaxation rate (R1, s^−1^) 30 min after injection of an albumin-binding contrast agent (gadofosveset trisodium). This technique has also been used successfully to quantify changes in permeability in the murine brachiocephalic artery after therapeutic intervention [111, 116]. More recently, Phinikaridou et al. [117] used this same technique to quantify endothelial permeability in aortic plaques in atherosclerotic rabbits, and demonstrated higher R1 (indicative of higher permeability) in aortic segments more prone to disruption after injection of Russell’s viper venom. Unlike quantitative dynamic imaging with DCE-MRI, these techniques measure signal enhancement or quantify tissue relaxation time only at a fixed point after contrast agent injection. This approach is particularly well suited for higher molecular weight or albumin-binding contrast agents, whose plaque uptake kinetics are intrinsically slower. While failing to capture the dynamic uptake of contrast agents over time, these techniques offer a simpler and robust alternative to DCE-MRI for quantification of plaque microvascularization and permeability.

## Flow-derived biomechanical wall parameters

### Wall shear stress

Atherosclerosis originates predominantly at regions with perturbed flow that can occur at the outer edges of vessel bifurcations. In these regions, hemodynamic wall shear stress (WSS), the frictional force sensitized by endothelial cells forming the inner lining of blood vessels, is weaker than in protected regions and can even exhibit direction reversal. The atherogenic endothelial phenotype resulting from low WSS mediates recruitment and activation of monocytes, which can subsequently lead to plaque formation [118]. WSS is also known to increase with increasing blood flow. In response, the vessel dilates to reduce blood flow such that WSS returns to normal values. Regions where WSS is chronically elevated, such as the apices of bifurcations in the cerebral vasculature, are predisposed to the formation of aneurysms [119]. WSS therefore represents a key link between blood flow and alterations in biomechanical vessel wall parameters.

Flow measurement using MRI has traditionally been performed using 2D phase-contrast imaging in most vascular beds. With gating to the cardiac cycle, a time-resolved (cine) measurement can be performed [120]. Currently, a comprehensive assessment of flow in an entire 3D volume (3D cine phase-contrast MRI or 4D flow MRI) is feasible [121], enabling the measurement of full time-varying 3D velocity fields in a wide variety of cardiovascular regions [122].

The importance of WSS in vascular disease has led to widespread interest among researchers in obtaining reliable estimates of WSS from MRI-measured velocity data. Oshinski et al. were the first to develop a method based on linear fitting through velocity values to obtain the velocity derivative (the shear rate) at the wall [123]. Multiplication of the shear rate by dynamic viscosity yields the WSS. Other groups developed WSS estimation based on parabolic fitting, which showed greater accuracy than linear fitting [124, 125]. Stalder et al. used cubic B-splines to derive the shear rate at the wall [126]. Another important hemodynamic parameter shown to correlate with atherosclerosis is the oscillatory shear index (OSI) [127]. OSI represents the temporal oscillation of WSS during the cardiac cycle, the deviation of WSS from its predominant direction parallel to the vessel. Thus, the OSI can be calculated using methods to estimate time-resolved WSS.

These techniques applied to MRI-measured flow data have provided valuable insight into the relation between abnormal WSS and pathophysiology. Duivenvoorden et al. showed that WSS was an independent predictor of carotid wall thickness, lumen area and vessel size [128]. Mutsaerts et al. found that WSS was associated with periventricular white matter lesions and cerebral infarcts [129]. Markl et al. observed that low WSS and high OSI, which are potentially atherogenic wall parameters, were predominantly concentrated at the posterior wall of the internal carotid artery in normal controls, a region known to be prone to atherosclerosis [130]. Wentzel et al. reported that the presence of atherosclerotic plaques in the descending aorta was associated with low WSS [131]. Other studies showed that WSS on the ascending aorta was elevated compared to healthy controls in bicuspid valve disease, which implicates a relationship between elevated WSS and aortic dilation [132, 133].

**Fig. 8:**
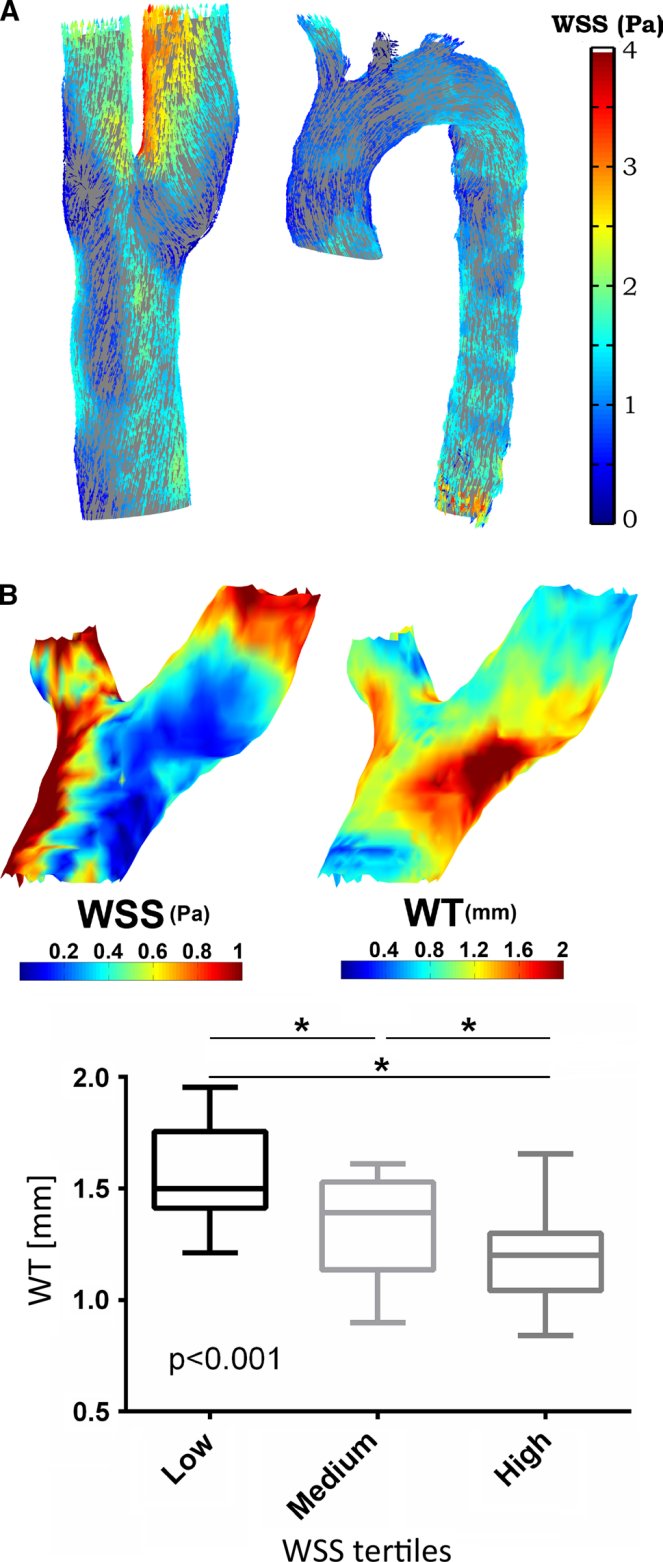
**a** Peak systolic WSS vectors along the carotid (*left*) and thoracic aorta (*right*). Adapted from Potters et al. [135]. **b** Example of a 3D wall thickness (WT [mm]) map of the carotid bifurcation and corresponding time-averaged 3D wall shear stress WSS (Pa) map of the same subject (top). WT was significantly different in each tertile and the highest WT was found in the lowest WSS tertile (bottom). Adapted from Cibis et al. [136]

However, these algorithms were based on 2D phase-contrast MRI or the manual placement of planes in 4D flow MRI data perpendicular to the vessel of interest. With 2D techniques highly focused on specific vascular landmarks, focal abnormal WSS expression on the vessel of interest may be missed. Also, the manual placement of 2D planes for WSS analysis can be a laborious task. Bieging et al. were the first to develop a WSS algorithm based on 4D flow MRI that was capable of estimating WSS along the entire wall of the ascending aorta [134]. The linear least squares method was used to fit a line through three velocity vectors along the inward normal. Potters et al. expanded on this method with the use of smoothing spline fitting to mitigate the influence of noise. The feasibility of estimating WSS along the entire aorta (the arch and descending part included) and the carotid bifurcation was also demonstrated (see Fig. 8a [135]). Cibis et al. recently found an inverse relationship between wall thickness and time-averaged WSS in patients with asymptomatic plaque, calculated with the algorithm described in Potters et al. in the carotid bifurcation (Fig. 8b) [136].

Some important considerations should be kept in mind when estimating 4D flow MRI-derived WSS. First, several studies showed that an accurate definition of the vessel wall is paramount [125, 135]. Nonetheless, low inter-observer variability in WSS was found for both 2D (<10%) and 3D (<5%) WSS algorithms [137, 138]. Second, the absolute value of WSS decreases with spatial resolution [126, 135]. Thus, 4D flow MRI-derived WSS is always underestimated compared to computational fluid dynamics where fine meshes are used [139]. Qualitatively, however, regions of high and low WSS and the direction of WSS tend to correspond well [140–142](Fig. 7).

### Arterial stiffness

Another parameter that can be assessed using phase-contrast MRI is pulse wave velocity (PWV), which characterizes the speed of the arterial pulse through a specific part of the circulation. PWV is a measure of arterial stiffness and can be directly correlated with the vessel wall elastic modulus *E* using the Moens-Korteweg equation:

$${\text{PWV = }}\sqrt {\frac{Eh}{\rho d}},$$

where *h* is the vessel wall thickness, *ρ* is the blood density and *d* is the vessel diameter. The elasticity of the vessel wall decreases as part of natural aging. MRI-based PWV measurements have indeed shown significant increases in PWV as a function of age in the carotid arteries and the aorta [143], ranging from roughly 5 m/s for young adults to 7–8 m/s in the elderly. More importantly, arterial stiffness has also been associated with atherosclerosis [144], and studies have shown that the measurement of PWV in various vascular beds significantly improves the prediction of future cardiovascular events [145–147].

**Fig. 9:**
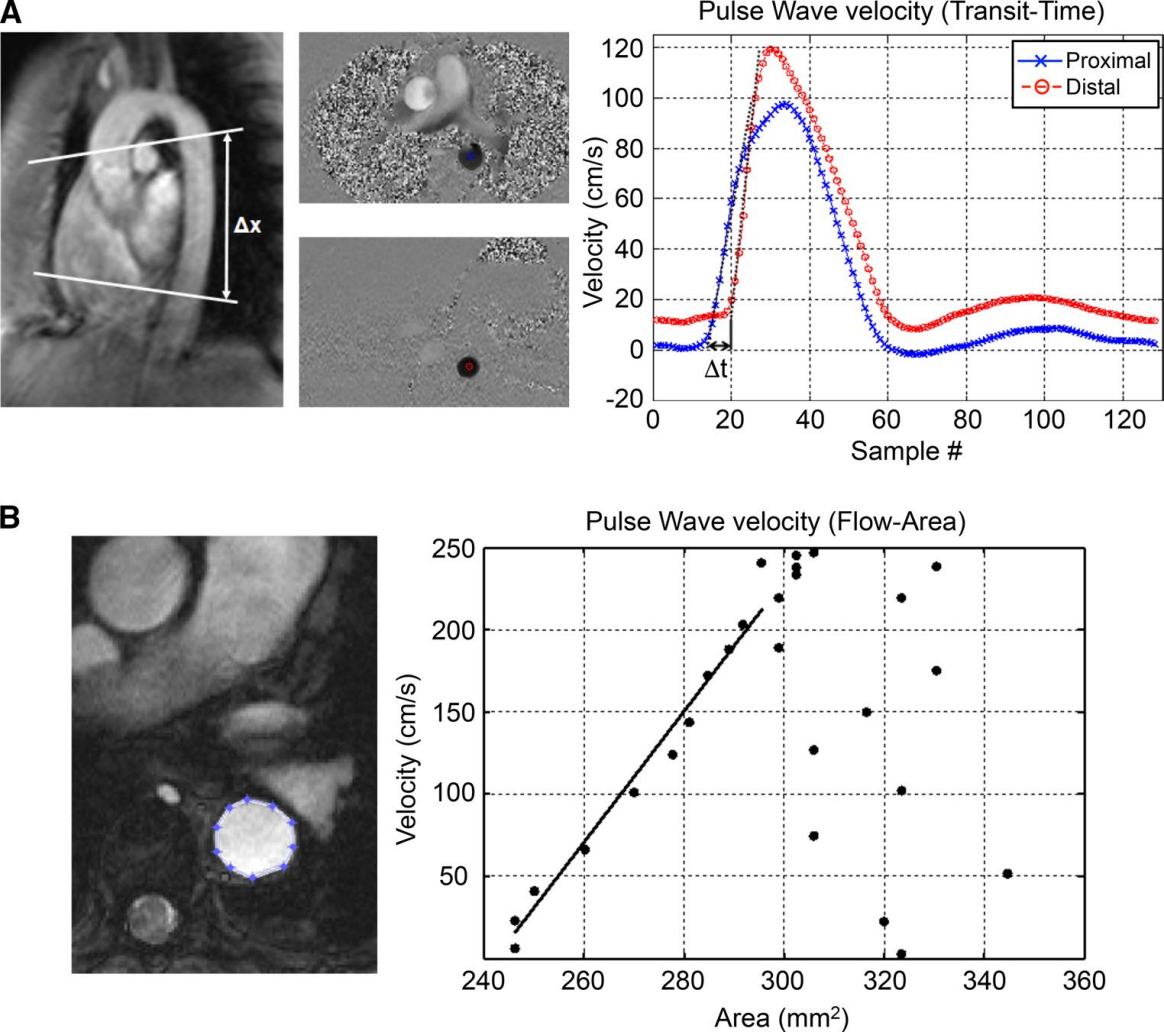
Different techniques for determing pulse wave velocity (PWV) in the aorta. **a** foot-foot method. The path length between two slices perpendicular to the aorta is divided by the time difference between the initial upslopes of the two flow curves. **b** QA-method. Lumen area is plotted as function of flow during the cardiac cycle. PWV is defined as the change in lumen area (A) divided by the change in flow (Q) during the linear portion of the QA-plot. Adapted from [148]

While Doppler ultrasound is the clinical workhorse for determining PWV, MRI might offer several advantages. Particularly in 3D anatomical imaging, MRI enables better visualization of each vessel segment, independent of angle and depth. This likely improves reproducibility due to ease of planning, as well as by a more accurate determination of the path length of the pulse wave [148, 149]. Many approaches to calculate PWV are mentioned in the literature and unfortunately no clear consensus exists on which method produces most reliable PWV values. The most widely used method is by measuring flow-time curves at two different slices along the vessel. The so-called foot–foot method can then be used to calculate PWV by dividing the path length between the slices by the time difference between the initial up-slopes of the two flow curves (Fig. 9a). The exact location of the foot is slightly affected by the baseline definition and the number of points used to define the linear part of the up-slope (usually 20–80% of the maximum flow). Another analysis method finds the time shift at which both curves have the highest correlation; however, this can be affected by the correlation window and presence of wave reflections starting from the systolic downslope [150]. Instead of relying on phase-contrast acquisitions in two slices, PWV could be calculated from measurements in a single or multiple oblique sagittal slices [151, 152]. Flow–time curves could then be plotted for each pixel along a centerline of the vessel, which produces a set of time shifts as a function of distance, presumably resulting in a more reliable estimate of PWV. For the aorta, this has been extended to volumetric flow measurements using highly accelerated 4D flow acquisitions, which further facilitates the planning of the imaging volume (see [153], Fig. 8). Tortuous vessels such as the carotids could benefit from this methodology, although temporal and spatial resolution still appear to be too low for this specific application. A third method provides even more local vessel wall PWV values by assessing changes in lumen diameter (A) as a function of flow (Q) at different time points during the cardiac cycle, where PWV is given by ΔQ/ΔA during the initial up-slope of this relation (Fig. 9b). In a study comparing the above-mentioned methods for the aorta, Ibrahim et al. found the best agreement between the foot–foot and cross-correlation methods, which also showed the highest reproducibility [148]. While analytical methods vary widely among studies, results for group-averaged aortic PWV have been quite consistent (4–5 m/s) in young adults [149–151, 154]. Unfortunately, less data is available on carotid PWV (~5.5 m/s) and femoral arteries (~7 m/s) [143, 155], for which the latter interestingly seems to decrease with age. Finally, while repeated measurements on the same day have shown good repeatability [149, 156], day-to-day physiological variations in blood pressure and blood flow might limit the applicability of PWV measurements for sensitive monitoring of gradual changes over time.

## Summary and future perspectives

In the last decade, technological advances have strengthened the position of MRI in the assessment of quantitative, physiological parameters regarding tissue structure and pathology. 3D blood suppression techniques and 3D time-resolved (4D) imaging have enabled the development of strategies for assessing the anatomical, structural and functional status of the vessel wall. A few consensus statements have been published on specific applications discussed in this review [157, 158]. Similar publications on “best practice” vessel wall imaging protocols are needed, and will help to propel this novel research field forward. In this respect, reproducibility studies on quantitative vessel wall T1/T2^(*)^, DCE and flow imaging protocols are highly important.

Aside from what is presented in this review, the search continues for MR techniques that quantify other specific markers related to vessel wall pathology, e.g. strain [159], or other contrast mechanisms to increase sensitivity for specific atherosclerotic plaque features. The latter might include T1_rho_ imaging for assessment of fibrosis [160] or susceptibility-weighted imaging to detect calcifications [161]. While there is much interest in techniques that do not rely on the use of MRI contrast agents, this review has shown the relevance of DCE imaging for measuring plaque microvascular volume and permeability, which are strongly associated with vessel wall inflammation. The use of untargeted iron oxide nanoparticles has also been briefly discussed here. Novel “smart” nanoparticles that specifically target biomarkers of inflammation have yielded very promising results in animal models of atherosclerosis [162–164]. By labeling with MRI contrast agents, accumulation of these nanoparticles could be quantified using T1 and/or T2 mapping protocols as described in this review. MRI of nuclei other than ^1^H, such as ^19^F, is also a topic of active investigation for the absolute quantification of plaque inflammation using perfluorocarbons [165].

Quantifying physiologically relevant parameters in addition to standard anatomical imaging naturally entails longer acquisition times and/or reduced SNR. Furthermore, several sources of errors, such as the choice of fitting algorithm, motion artifacts, and data SNR, may affect parameter quantification. Aside from developing new quantitative readouts, current efforts are predominantly focused on improving the accuracy, precision and scan efficiency of existing methods by making use of ultra-high-field imaging as well as novel reconstruction algorithms.

### 7T MRI

Recent years have seen the introduction of 7T imaging in clinical research, with the direct advantage of an increase in SNR directly proportional to the strength of the magnetic field [166].

**Fig. 10:**
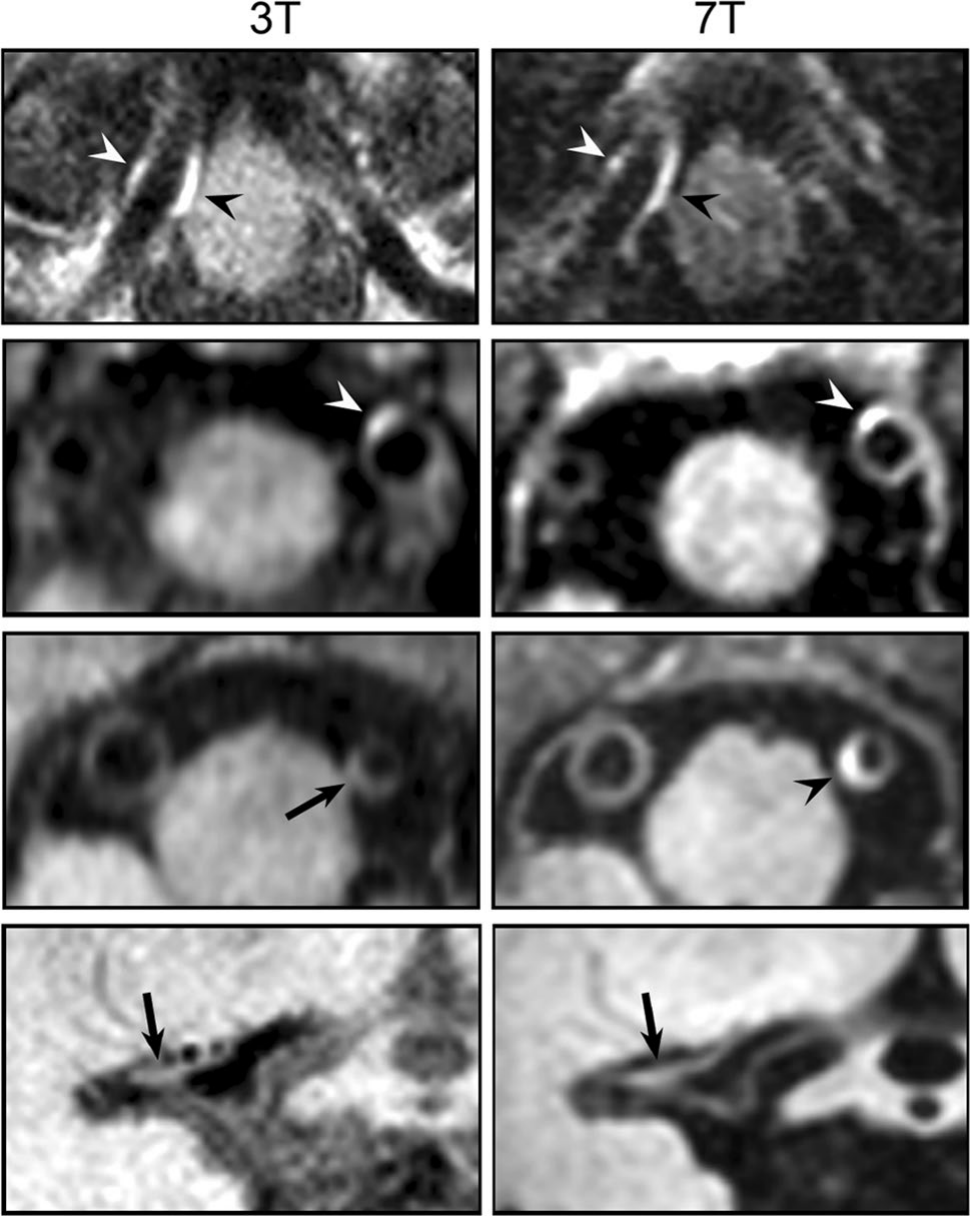
Comparison of post-contrast intracranial vessel wall imaging using 3T and 7T MRI. 7T MRI largely resulted in enhanced vessel wall delineation (due to enhanced CSF suppression) and more frequent detection of atherosclerotic lesions that were missed at 3T (*rows 3 and 4*). Depicted are the right proximal artery (*row 1*), left proximal vertebral artery (*rows 2 and 3*) and M1 segment of the middle cerebral artery (*row 4*). Adapted from Harteveld et al. [18]

While challenging at 3T [167], this makes 7T MRI of particular interest for characterization of intracranial atherosclerosis, where the even smaller vessel size requires sufficient signal for accurate delineation of the vessel wall. Researchers from UMC Utrecht have been pioneers in this field by developing 7T intracranial vessel wall imaging protocols [168], as well as evaluating the benefit of 7T versus 3T MRI in detecting atherosclerotic plaques (see also Fig. 10) [18]. On the one hand, they found that 7T MRI resulted in better wall definition. This was mainly achieved by exploiting the increased SNR for higher acceleration factors, enabling the use of very robust but inherently time-inefficient inversion recovery-based cerebrospinal fluid (CSF) suppression. Although in many cases lesions were detected only at 7T, the authors also noted a significant number of cases showing the opposite. Similarly, studies to date on carotid plaque characterization have shown no strong indications of the superiority of 7T over 3T [169, 170], which might be related to the more inhomogeneous transmit field at 7T compromising non-selective blood suppression techniques, as well as signal homogeneity. In this respect, there is a considerable need for better-designed high-field carotid coils in order to achieve improved transmit and receive characteristics [171, 172]. Similarly, developments in cardiac 7T coil design may boost the field of coronary imaging by exploiting the increase in SNR for obtaining higher spatial resolution [173].

### Advanced reconstruction techniques

Quantitative imaging generally comes with the need to acquire multiple images, either having different sensitivity to the parameter of interest (T1, T2, ADC) or sampling a dynamic process over time (DCE, flow). Long acquisition times are therefore one of the big hurdles in this field, and complicate the translation of such methods to a clinical environment. What may be able to change this in the near future is the rapid improvements in reconstruction and post-processing techniques. In recent years, mathematics has played an increasingly important role in advancing MRI technology. In particular, the introduction of “compressed sensing” has taught us that images can be reconstructed with far less data than was considered necessary using assumptions of image sparsity in combination with iterative reconstruction algorithms [174]. As this is quite a generally applicable concept, many researchers have already experimented with compressed sensing to improve vessel wall MRI. For example, 3D MSDE acquisitions were accelerated to a factor of 5 without significant deviations in assessing vessel wall or plaque component dimensions [175, 176]. Gong et al. realized that multi-contrast vessel wall MRI could be significantly accelerated by assuming significant shareable information between the different contrast acquisitions [177]. The use of their proposed reconstruction algorithm allowed for highly accelerated T2w and PDw scans (up to a factor of 6) once a moderately accelerated T1w scan (e.g. using SENSE) was available. Using regular CS techniques, Yuan et al. reduced a 3D MSDE-based T2 mapping protocol to a clinically acceptable imaging time of 7 min [178]; reconstruction of separate images was done independently. A more promising technique related to quantitative relaxation time (T1/T2) mapping are model-based algorithms that use prior knowledge of the relaxation equations to reconstruct undersampled scans at multiple inversion or echo times [179, 180]

For dynamic or time-resolved imaging, temporal relations between images are used in order to achieve high undersampling factors for the individual time frames. This technique shows great promise for achieving higher-temporal-resolution 3D DCE imaging [86] or allowing interleaved sampling of black- and bright-blood images for the simultaneous measurement of the arterial input function and vessel wall signal response [106]. For 4D flow applications, the use of k-t GRAPPA has enabled acceleration factors of up to 5 without substantial errors in derived WSS parameters [181].

Perhaps the greatest benefit of freely undersampling k-space data is the ability to select the optimal data retrospectively. This can be used to circumvent the need for cardiac and respiratory gating, without losing scan efficiency, and order the data in the reconstruction process based on available data of cardiac and respiratory motion from either sensors or MR navigators. Using appropriate motion correction algorithms, this even allows for 100% scan efficiency [182, 183]. Recent impressive data from Ginami et al. [184] showed high-quality coronary vessel wall imaging by making reconstructions using different timings of the acquisition window within the cardiac cycle in order to retrospectively achieve the optimal vessel wall delineation.

### Multimodal imaging: PET/MRI

In addition to the development of novel MR methods and contrast mechanisms, the integration of MRI with other imaging modalities that interrogate different aspects of plaque physiology is quickly becoming a reality. As a notable example, the introduction of simultaneous PET/MRI systems allows for the seamless combination of anatomical and physiological imaging with MRI, and metabolic/functional imaging with PET. PET is a highly sensitive modality, and has already been extensively validated for quantification of plaque macrophages with ^18^F-FDG [185, 186]. However, PET is an imaging modality with intrinsically low spatial resolution, and it is traditionally combined with computed tomography (CT) for improved anatomical localization. Combining PET with MRI instead of CT offers several advantages. MRI provides high-spatial-resolution imaging and better soft tissue contrast than CT, and enables the quantification of several physiological parameters in the vessel wall, as previously described in this review. The better anatomical definition of MRI can be used to apply partial volume and motion corrections to improve the localization of the PET signal [187, 188].

Last but not least, combining PET with MRI instead of CT reduces patient exposure to ionizing radiation—a highly desirable feature for longitudinal, repeated imaging in patients with chronic diseases (such as atherosclerosis). The fact that the two modalities are intrinsically co-registered allows for easier image interpretation, image analysis and experimental design [188, 189]. However, there are also specific challenges that may arise when combining these two modalities, such as the effective conversion of MR images to accurate PET attenuation maps, which are still the subject of active investigation [189].
